# Supporting lifestyle change in obese pregnant mothers through the wearable internet-of-things (SLIM) -intervention for overweight pregnant women: Study protocol for a quasi-experimental trial

**DOI:** 10.1371/journal.pone.0279696

**Published:** 2023-01-19

**Authors:** Johanna Saarikko, Anna Axelin, Emilia Huvinen, Amir M. Rahmani, Iman Azimi, Miko Pasanen, Hannakaisa Niela-Vilén

**Affiliations:** 1 Department of Nursing Science, University of Turku, Turku, Finland; 2 Department of Obstetrics and Gynaecology, Turku University Hospital and Faculty of Medicine, University of Turku, Turku, Finland; 3 Department of Obstetrics and Gynaecology, University of Helsinki and Helsinki University Hospital, Helsinki, Finland; 4 School of Nursing, University of California Irvine, Irvine, California, United States of America; 5 Department of Computer Science, University of California Irvine, Irvine, California, United States of America; 6 Department of Future Technologies, University of Turku, Turku, Finland; GERMANY

## Abstract

**Objectives:**

To assess, in terms of self-efficacy in weight management, the effectiveness of the SLIM lifestyle intervention among overweight or obese women during pregnancy and after delivery, and further to exploit machine learning and event mining approaches to build personalized models. Additionally, the aim is to evaluate the implementation of the SLIM intervention.

**Methods:**

This prospective trial, which is a non-randomized, quasi-experimental, pre-post intervention, includes an embedded mixed-method process evaluation. The SLIM Intervention is delivered by public health nurses (n = 9) working in maternity clinics. The public health nurses recruited overweight women (n = 54) at their first antenatal visit using convenience sampling. The core components of the intervention i.e. health technology, motivational interviewing, feedback, and goal setting, are utilized in antenatal visits in maternity clinics starting from gestational week 15 or less and continuing to 12 weeks after delivery. Mixed effect models are used to evaluate change over time in self-efficacy, weight management and weight change. Simple mediation models are used to assess calories consumed and moderate to vigorous physical activity (MVPA) as mediators between self-efficacy and weight change. Signal processing and machine learning techniques are exploited to extract events from the data collected via the Oura ring and smartphone-based questionnaires.

**Discussion:**

The SLIM intervention was developed in collaboration with overweight women and public health nurses working in maternity clinics. This study evaluates the effectiveness of the intervention among overweight women in increasing self-efficacy and achieving a healthy weight; thus, impacting the healthy lifestyle and long-term health of the whole family. The long-term objective is to contribute to women’s health by supporting weight-management through behavior change via interventions conducted in maternity clinics.

**Trial registration:**

The trial was registered at the Clinicaltrials.gov register platform (ID NCT04826861) on 17 March 2021.

## Introduction

Obesity has nearly tripled during the last few decades [1] and has become a global health concern; this trend can also be seen among pregnant women. In Finland, for example, 41.7% of pregnant women were overweight (BMI ≥25) and 17.6% were obese (BMI ≥30) in 2019 [2]. Correspondingly, almost a third (29%) of pregnant women in the US were obese in 2019 [3]. Maternal overweight increases the risks for obstetric complications and newborn mortality and morbidity, such as gestational diabetes, obstetric intrapartum complications, and caesarean section, macrosomia and stillbirths [2, 4, 5]. Consequently, preventing and decreasing obesity in pregnant women is essential to improve the health of future generations. Pregnancy is an exceptional period in a women’s life; there is often a shift in attitude and a spontaneous change in positive health behaviors, making women more receptive to advice on nutrition and physical activity. It is crucial to support their willingness to change health behaviors and encourage the motivation to take care of their own and their unborn child´s health. Healthy lifestyle behaviors adopted during pregnancy are also likely to have positive impacts on women’s health after pregnancy [6].

Previous behavioral lifestyle interventions focusing on a healthy diet and physical activity have succeeded in reducing gestational weight gain and improving pregnancy outcomes, for example, by reducing the number of macrosomia or caesarean sections. Further, some interventions have reduced neonatal respiratory distress syndrome and some interventions have also been associated with a reduction of postnatal depression [7–10]. However, these interventions have focused on single outcomes, and are not established as part of maternity care. In addition, counseling that focuses on giving general information on healthy diets and exercise is not usually effective; instead, the focus should be placed on individual barriers and challenges. Furthermore, weight gain may not be the most optimal parameter to focus on during pregnancy because obesity is a sensitive and stigmatizing subject, which is often found to be difficult to discuss with pregnant women. Hence, the focus of interventions should be on health promotion instead of weight loss [11]. Overall, there is a clear necessity for innovative and cost-effective lifestyle interventions to promote weight-management and health outcomes [8, 12].

As an alternative to teaching obese pregnant women to eat healthily and increase their activity the target should be to enhance the individual’s self-efficacy, which is a crucial psychosocial factor underpinning change in behavioral practices [13, 14]. In addition, self-efficacy has been shown to be a strong predictor of health behaviors and weight control, including physical activity and dietary intake and therefore, body weight [15, 16]. More comprehensive research is required to investigate methods of incorporating improvement in the self-efficacy of overweight women during pregnancy and the postpartum period. These methods would need to help in overcoming barriers to weight management and provide the means to implement an optimal weight-management intervention. E-Health applications have been shown to be effective alternatives to standard practice; these apps and exercise programs supporting weight management have indicated a tendency, for example, toward less gestational weight gain, and an increase in step [17, 18]. In addition, continuous monitoring has been shown to be a good method for women and their health care professionals to create personalized interventions that minimize weight gain and help women to adopt healthy lifestyles during pregnancy.

Pregnant women meet health care professionals regularly, which enables the development of continuous collaboration and support between healthcare professionals and pregnant women. Therefore, pregnancy is an optimal period for establishing weight management interventions, which are most effective when introduced as part of routine antenatal care [11, 19]. Previous interventions have not been implemented as apart of maternity care. To implement interventions effectively the focus should be on the needs of overweight women and the promotion of self-efficacy. In the context of pregnant women, it is therefore important to understand the barriers and enablers that can prevent or help achieve changes in health outcomes. Given the importance of effective weight-management interventions, there is a need to develop feasible implementation strategies to support the adoption of interventions in real life settings. Local content and tailored implementation strategies could be crucial to bridging the gap between implementation research and clinical practice [20, 21].

The focus of the SLIM intervention is on supporting and refining existing antenatal services, which have proven to be effective [22]. The SLIM intervention offers health data to be utilized in maternity care: public health nurses (PHN) give feedback and set goals in collaboration with overweight pregnant women using motivational interviewing methods. The SLIM intervention has been initiated in close collaboration with the end users, i.e. overweight pregnant women and PHNs working in maternity clinics, and its target is to enhance the participants’ self-efficacy and to promote their weight management. The effectiveness and implementation will be evaluated in routine maternity care using implementation framework [23].

### Aims

The first aim of this study is to assess the effectiveness of the SLIM intervention in terms of improving self-efficacy in eating and physical activity (primary outcome) and preventing excessive gestational weight gain (secondary outcome). Further, the events and patterns from the collected health parameters during pregnancy and the postpartum period will be extracted and the variations and trends will be tracked to build personalized models. The second aim is to evaluate the implementation of the SLIM intervention with regard to perceived fidelity, acceptability, appropriateness, and feasibility among PHNs and women with overweight or obesity.

## Methods

### Design and settings

This is a prospective intervention trial that is non-randomized and quasi-experimental (pre-post) and includes an embedded mixed-method process of evaluation. The study will be conducted in two public maternity clinics located in four municipalities in the Hospital District of Southwest Finland between April 2021 and May 2023.

In Finland, pregnant women receive free care from public maternity clinics as part of the general health care arranged by municipalities. Women usually visit a PHN a total of 10–11 times during pregnancy and after delivery. Public health nurses in maternity clinics provide support with particular attention to the promotion of welfare and health. This includes guidance and support regarding sleep, physical activity and nutrition and food recommendations for pregnant and breastfeeding mothers [24]. With reference to maternal weight gain during pregnancy, PHNs also provide advice on weight management based on pre- pregnancy BMI [6].

#### Target group 1: Overweight women

Overweight women (n = 54) were recruited at their first antenatal visit using convenience sampling. The power analysis was calculated based on two scales, self-efficacy in eating (WEL) and physical activity (PASE). Calculations were done with one sided t-tests with an effect size of 0.50, a power level of 0.80 and a significance level of 0.05. A 20% loss was included in the calculation. Based on both scales, a sample of 54 overweight pregnant women was needed to detect the difference in changes in eating self-efficacy or self-efficacy in physical activity.

Women were eligible for inclusion if they were 1) pregnant ≤15 weeks of gestation, 2) ≥18 years of age and 3) overweight or obese (BMI ≥ 25kg/m2) and 4) had adequate language skills in Finnish. Women were excluded if they 1) did not have a mobile device to download applications and synchronize data, 2) had a diagnosis of severe mental illness, 3) were diagnosed diabetes type 1 before pregnancy and/or 4) had mobility limitations.

#### Target group 2: Public health nurses

All PHNs (n = 9) working in selected maternity and child health clinics were eligible for the study. The researcher applied for approval from the Nursing Director of the health center and contacted the PHNs via e-mail and scheduled meetings. Public health nurses were already recruited in the intervention development phase.

### Intervention

**Fig 1:**
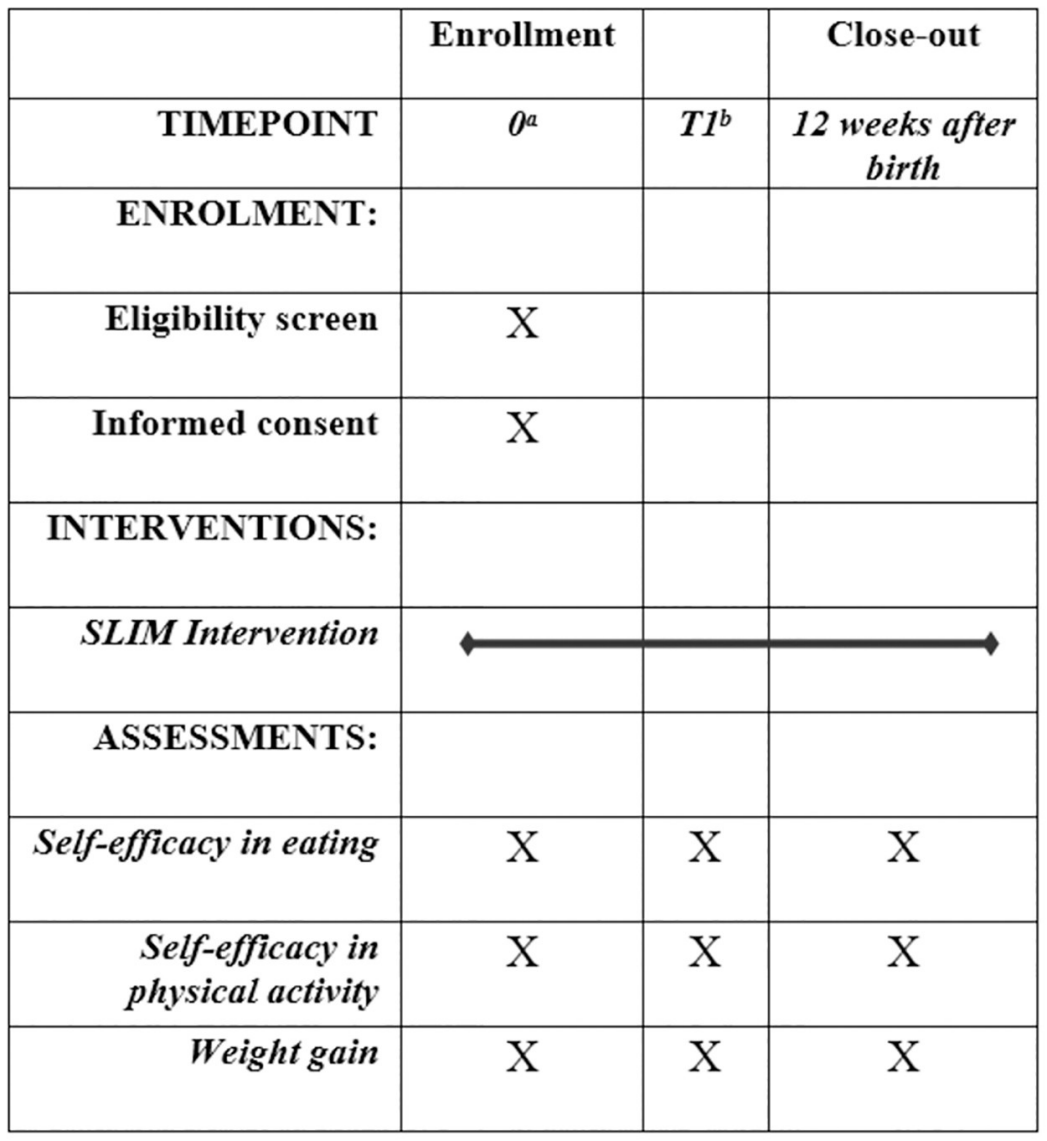
SPIRIT schedule presenting participant timeline. ^a^ Pregnancy week less than 15. ^b^ Pregnancy week 34.

The SLIM intervention is targeted at overweight pregnant women with a goal to improve their self-efficacy in weight management. The intervention is being delivered by PHNs working in maternity clinics (Fig 1).

### Development of the intervention

**Fig 2:**
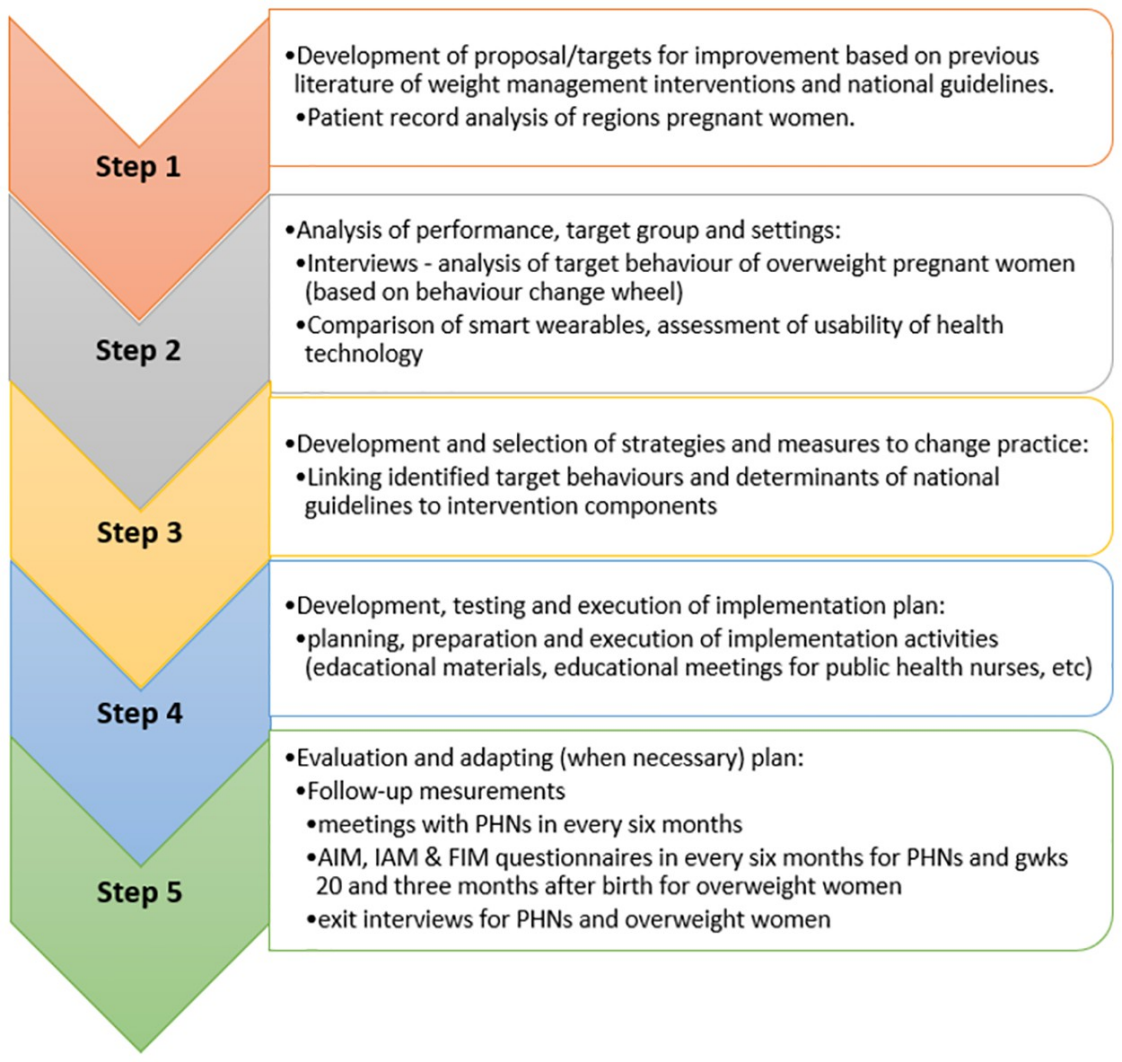
The development process of the weight management intervention for overweight pregnant women.

The Grol and Wensings [25] five-step implementation-of-change model guided the development process (Fig 2) and overweight pregnant women and PHNs working in selected maternity clinics participated in the development of the intervention.

**Step 1**: Development of proposal/targets for improvement were conducted based on previous literature of weight management interventions, national guidelines and local patient record data. Data on pregnancy and delivery, BMI before pregnancy and after delivery, breastfeeding information and depression scores were obtained from the electronic patient records collected in 2018 at one maternity clinic. In order to establish a detailed understanding of the weight management problem, these acquired statistics for the region’s overweight women and the information on obesity and weight management interventions based on the literature [9, 26, 27] were introduced to PHNs (n = 8) and their Nursing Director. A total of 84 pregnant women attended pre-pregnancy care in maternity clinics in 2018 in the area of the two municipalities and the majority (67.9%) were overweight or obese.

**Step 2**: The analysis of performance, the target group and settings were examined through individual interviews with overweight women (n = 11) and focus group interviews with PHNs (n = 5) [28]. Weight management behaviors were identified from the perspective of the maternity care professionals and the overweight and obese women with the help of the Behaviour Change Wheel (BCW) [29]. The results revealed that interventions should offer clear advice and non-judgmental support during pregnancy and after delivery by targeting women’s capabilities, opportunities, and motivation. In addition, health technology could be a valuable component of the intervention, as well as an implementation strategy that would provide ways to approach this topic and support women in maternity clinics. To assess the usability of health technology and wearable devices as a potential intervention element, a purposive sample of overweight and obese women (n = 7) and PHNs (n = 6) wore smart wearables for seven consecutive days. Participants completed a semi-structured diary each day to assess the comfort and usability of the devices and the application. Participants preferred the Oura Smart Ring to the Samsung smart watch, as it was comfortable to wear and had a beautiful appearance.

**Step 3**: Development and selection of strategies and measures for practicing change were conducted by linking identified target behaviors and determinants of the national guidelines to the intervention components. Previously identified weight management target behaviors [28] were mapped to the matrix of BCW (S1 Table). These results were presented to the PHNs and based on their discussions and consensus the most helpful elements for their clinical work in maternity clinics were chosen. These elements were *health technology* to support self-monitoring and goal setting, *motivational interviewing*, *feedback and goal setting* to enhance the motivation to improve self-efficacy in weight-management. A template for the intervention description and replication (TIDieR) was used as a guideline to improve the comprehensive reporting of the intervention (S2 Table).

**Step 4**: Development, testing and execution of the implementation plans were conducted in collaboration with PHNs. Meetings were conducted with PHNs to present a summary of the previous phases of the study and the interventions core components based on TIdieR. In addition, more information on specific requirements as regards the implementation of the intervention were collected from PHNs; these were used to choose strategies to support the implementation of the SLIM intervention in the daily work of PHNs. The following are the multi-faceted implementation strategies which were chosen and classified using the Expert Recommendations for Implementing Change (ERIC) [30]: *educational meetings*, *educational materials*, *local opinion leaders*, *mandate change and reminders*. Before the recruitment of the overweight pregnant women, all eligible PHNs were invited to attend *educational meetings*, which involved one-hour sessions in scheduled appointments and were delivered by the researchers. In addition, *educational materials* developed by researchers were delivered during the meetings. During the data collection period, researchers scheduled workshops with maternity clinic nurses every six months. *The local opinion leaders* were selected from among the PHNs, one in each maternity clinic. These leaders provided information, and played an important role in liaising with other nurses delivering intervention and also influenced their participation. *Mandate change*, requiring leaders to declare the importance of the innovation and their determination to have it implemented was conducted every two months in regular meetings of the maternity clinic staff. *Reminders* were sent to PHNs via email every month. To promote adherence to the intervention nurses were encouraged to contact the researchers if any issues related to the implementation of the intervention occurred. The specifications of the implementation strategies are presented in the supplementary material (S3 Table) [30].

**Step 5**: The evaluation plan for intervention implementation is presented below and in Fig 1.

### SLIM intervention

The core components of the SLIM intervention are *health technology*, *motivational interviewing*, *feedback and goal setting*. Health technology includes the Oura-ring, an electronic food diary and a SLIM application. The Oura ring is a commercial wearable ring that captures various health parameters and provides various daily scores for the user. The ring–equipped with different biosensors including a built-in photoplethysmography (PPG) sensor and an inertial measurement unit (IMU)–provides heart rate, nocturnal heart rate variability, nocturnal respiration rate, sleep duration, body temperature, and step counts. The ring is water resistant, lightweight and easy to use for short-term and long-term monitoring. The ring’s battery allows up to one-week data collection per charge. The collected data are transferred to the Oura mobile app (iOS or Android) via Bluetooth and then to the cloud server [31]. Participants wear the Oura rings continuously starting from gestational week 15 or less and continue until the postnatal visit 12 weeks after delivery. The electronic food diary, FatSecret is a free of charge, online diet, nutrition and weight loss service that allows users to set up unique user profiles, track their activity, food intake and weight. The services offered by FatSecret include the FatSecret websites and mobile applications [32]. Participants complete a food diary for one week at three timepoints: gestational week 15 or less, gestational week 34 and eight weeks after the delivery. The SLIM application is a platform that enables researchers to send e.g. questionnaires and surveys to participants. PHNs utilize the data monitored and collected with the Oura smart ring and the food diary during routine maternity care visits to evaluate physical activity, sleep and nutrition. PHNs give feedback to the women based on the data. They use motivational interviewing focusing on helping individuals to identify and resolve any ambivalence about changing behavior [33], and create goals in collaboration with women. The goals are documented in patient records. Participants are asked to answer questionnaires at three timepoints: gestational week 15 or less, gestational week 34 and 12 weeks after the delivery.

### Evaluation of the effectiveness of the SLIM Intervention

#### Data collection

In the initial antenatal appointment, the PHNs provide a letter of information and ask permission for the researchers to be in contact with the potential participants. The researchers then explain the study purpose and procedures to the women in a scheduled appointment. In addition, an Oura ring, and instructions on how to use it and other applications are given to each participant by the researchers after informed consent.

#### Outcome measures

The primary outcomes for the effectiveness of the intervention are self-efficacy in eating and physical activity and reduced weight gain during pregnancy (secondary outcome). To track variations and trends, health parameters are collected, these include: weight retention postpartum, depressive symptoms, quality of life, pregnancy specific anxiety, perceived stress, sense of coherence, eating behavior and lifestyle patterns, physical activity levels, sleep quality, stress levels and dietary intake. (Fig 1).

#### Measurements

Participants complete the questionnaires via the SLIM application at recruitment (<15 pregnancy week), at 34 gestational weeks and 12 weeks after delivery.

#### Primary outcomes

Eating self-efficacy and physical activity self-efficacy are measured with following questionnaires:

**Weight Efficacy Life-Style Questionnaire (WEL)**: is a standard, validated and clinically meaningful measure of eating self-efficacy. WEL consists of 20 items, which are scored on a Likert-type scale from 0 to 9 (0: Not confident, 9: Very confident). Answers are assembled into five factors (four questions for each factor): confidence in being able to resist the desire to eat in the following contexts: negative emotions, availability, social pressure, physical discomfort and positive activities. The eating self-efficacy score is calculated by adding together all 20 items. A level of eating self-efficacy is defined by a high score on the WEL questionnaire [34, 35]. The validation studies reported an internal consistency for Cronbach’s α of 0.70–0.90 in overweight Caucasian population.

**Self-Efficacy for Physical Activity Scale (PASE)** is a valid questionnaire to assess confidence in the ability to exercise when faced with specific barriers. It has been modified for pregnant women. Participants are asked to rate their confidence in engaging in physical activity across a variety of situations. Responses are measured on a Likert scale ranging from 1 to 5 (1 = *not at all confident*, 5 = *extremely confident*). The PASE score is calculated by taking the sum of the 5 items. The internal consistency for Cronbach’s α was between 0.70–0.78 in a Latino women sample [36, 37].

#### Secondary outcome

**Weight gain** is measured by PHNs at every antenatal appointment, on average eight times. The women’s weight before delivery is then compared to the national recommendations based on their BMI before pregnancy. For those women whose pre-pregnancy BMI is 25–29.9, the recommended weight gain during pregnancy is 7–11 kg and for those women whose pre-pregnancy BMI is 30 or more, a gain 5–9 kg weight during pregnancy is recommended [38].

#### Health parameters

**Edinburgh Postnatal Depression Scale (EPDS)** is a reliable and valid 10-item questionnaire that screens women for symptoms of emotional distress during pregnancy and the postnatal period. The total score is calculated by adding the numbers selected for each of the 10 items. Depression is defined by a high score on the EPDS. Cut-off scores range from 9–10 to 13–14 [39, 40]. In this study, a cut-off score ≥10 has been used to indicate possible depression. The reliability values of the Edinburgh Depression Scale indicated by Cronbach’s *α* coefficient per trimester were 0.82, 0.83, and 0.84, respectively among an unselected sample of 845 pregnant women [41, 42].

**WHOQOL-BREF** is a well-accepted, valid generic 26-item instrument and the mostly commonly used instrument to assess quality of life. Responses to questions are measures on a 1–5 Likert scale where 1 represents "disagree" or "not at all" and 5 represents "completely agree" or "extremely”. WHOQOL-BREF has four domains: Physical health, psychological health, social relationships and environment. The score is calculated by adding together the point values for the questions corresponding to each domain and then transforming the scores to a 0-100-point interval. The first two questions on the WHOQOL-BREF do not correspond to a domain, but are meant to provide a global assessment of quality of life. Higher scores in each of the domains correspond to a greater perceived quality of life. The internal consistency measured by Cronbach’s alpha ranged from 0.71 to 0.77 in the sample of 190 postpartum women [43, 44].

**PRAQ-R2** is a valid pregnancy-specific anxiety questionnaire. Responses are measured with a Likert type scale ranging from 1 (definitely not true) to 5 (definitely true). The items in the PRAQ-R2 can be ordered into three subscales: Fear of giving birth, worries about bearing a physically or mentally handicapped child and concern about one’s own appearance. Cronbach’s alphas across the total scale were good (above 0.80) in a sample of Finnish pregnant women (n = 1152) [45].

**Perceived Stress Scale (PSS-4)** is a validated measure which rates on a 5-point scale the extent to which participants have experience stress-related symptoms in the past month. Responses are measured with a Likert type scale ranging from 0 (never) to 4 (very often), the Cronbach’s α = 0.77 (English sample). Higher values indicate more stress [46, 47].

**Sense of Coherence (SOC-13)**: is measured with a 13-item SOC questionnaire, which has three subscales: Meaningfulness (4 items), Comprehensibility (5 items), and Manageability (4 items). Responses are scored on a 7-category semantic differential scale with two anchoring responses tailored to the content of each item. Five items (1, 2, 3, 5, and 7) are reversed before adding together the scores to obtain the total score. The total score can range from 13 to 91 and a higher score indicates a higher SOC. The internal consistency measured by Cronbach’s alpha ranged from 0.70 to 0.92 [48]. The sense of coherence scale shows high internal consistency [49].

**The three-factor eating questionnaire (TFEQ-R18)** is used to measure eating behavior tendencies [50], which has three domains: cognitive restraint (6 items), uncontrolled eating (9 items) and emotional eating (3 items). Responses are measured on a 4-point Likert scale, except for one item using an 8-point Likert scale. The range of total scores are from 0–100. To calculate the scores of the subscales, items 1–13 are reverse-coded, so that higher scores indicate a higher level of eating behavior tendencies. Cronbach’s alphas for the three domains were 0.71, 0.88 and 0.89 respectively (Swedish sample).

**6-FQ is** a 27-item self-administered instrument with psychometric properties that measures lifestyle pattern factors: Convenient Diner, Fast Pacer, Easily Enticed Eater, Exercise Struggler, Self-Critic and All-or-Nothing-Doer. Responses are measured on a 4-point Likert scale: “don’t agree at all” (0), “agree a little” (1), “agree” (2) and “strongly agree” (3). The score range is from 0–81, and a higher score indicates unhealthy lifestyle pattern factors. The Cronbach’s reliability estimates for internal consistency ranged from 0.76 to 0.85 (sample of overweight or obese adults) [51].

**Background information questionnaire**: The maternal background characteristics including age, BMI, marital status, education and employment status are collected with a self-administered background information questionnaire.

#### Patient record data

Data on pregnancy and delivery is comprised of gestational weight gain, postpartum weight retention, blood pressure, pregnancy complications, birth outcomes, newborn data such as birthweight and LGA. In addition, data is collected about several other maternal items: chronic diseases, psychological history, use of medications, diagnoses and complications in previous pregnancies (such as, GDM, preeclampsia, preterm delivery (<37 weeks of gestation) and miscarriage).

#### Well-being data

*Data on dietary intake* that is collected with the mobile app (FatSecret), and the nutritional intake (Calories, Protein, Fat and Carbohydrate) are both included in analysis. The app also displays the data by meal and snack eaten to observe meal patterns. FatSecret has been shown to be accurate and practical [52]. The outcome variable of interest in this study is the total kilocalories per day.

*Data on activity*, *sleep and stress* is collected with the Oura-ring. Studies in the literature have indicated the reliability of the ring in terms of sleep, physical activity, heart rate, and heart rate activity [53–55].

#### Data analysis

The data will be analyzed statistically. Appropriate descriptive statistics will be calculated for the background variables, WEL, PASE, weight change, EPDS, WHOQOL-BREF, PRAQ-R2, PSS-4, SOC-13, TFEQ-R18, 6-FQ, calories consumed, and the Ouras parameters. Variables like the EPDS, baseline BMI and Ouras wear time will be tested for their association with changes in self-efficacy in eating and physical activity. Mixed effect models will be used to evaluate change over time in self-efficacy in eating and physical activity and weight change. Simple mediation models will be used to assess calories consumed and MVPA as mediators between self-efficacy and weight change.

Machine learning and event mining approaches are being exploited in the intervention to build personal models–such as sleep and physical activity–for each individual. The relationships between the collected parameters, including weight gain, physical activity, sleep, and stress will also be investigated. Signal processing and (for example, unsupervised and semi-supervised) machine learning techniques are being exploited to extract events–i.e., high abstraction level information–from the data collected via the Oura ring and the questionnaires. These events characterize pregnant women’s daily life or lifestyle: e.g., their physical activity during the day or stress levels during sleep and these events are defined based on different attributes in certain epochs varying from minutes to a day. The events will be divided into three categories: activity, health, and context. Subsequently, the relationships between the events and their attitudes will be investigated via statistical analysis techniques. For example, when an undesired event (e.g., stress) is detected during pregnancy, statistical analysis techniques (e.g., Chi-Square test) will be used to evaluate the relationships between the event and the mother’s activity, health, and context. Moreover, higher data abstraction will be performed to record events in a chronological order. Data analytic techniques ranging from simple rule-based algorithms to machine learning classifications will be developed to extract patterns from the collected data and events. To this end, machine-learning-based methods (e.g., autoencoders) will be developed to build personalized models, by which the pregnant woman’s real state is compared with the desired state.

#### Process evaluation of the implementation of the SLIM intervention

**Fig 3:**
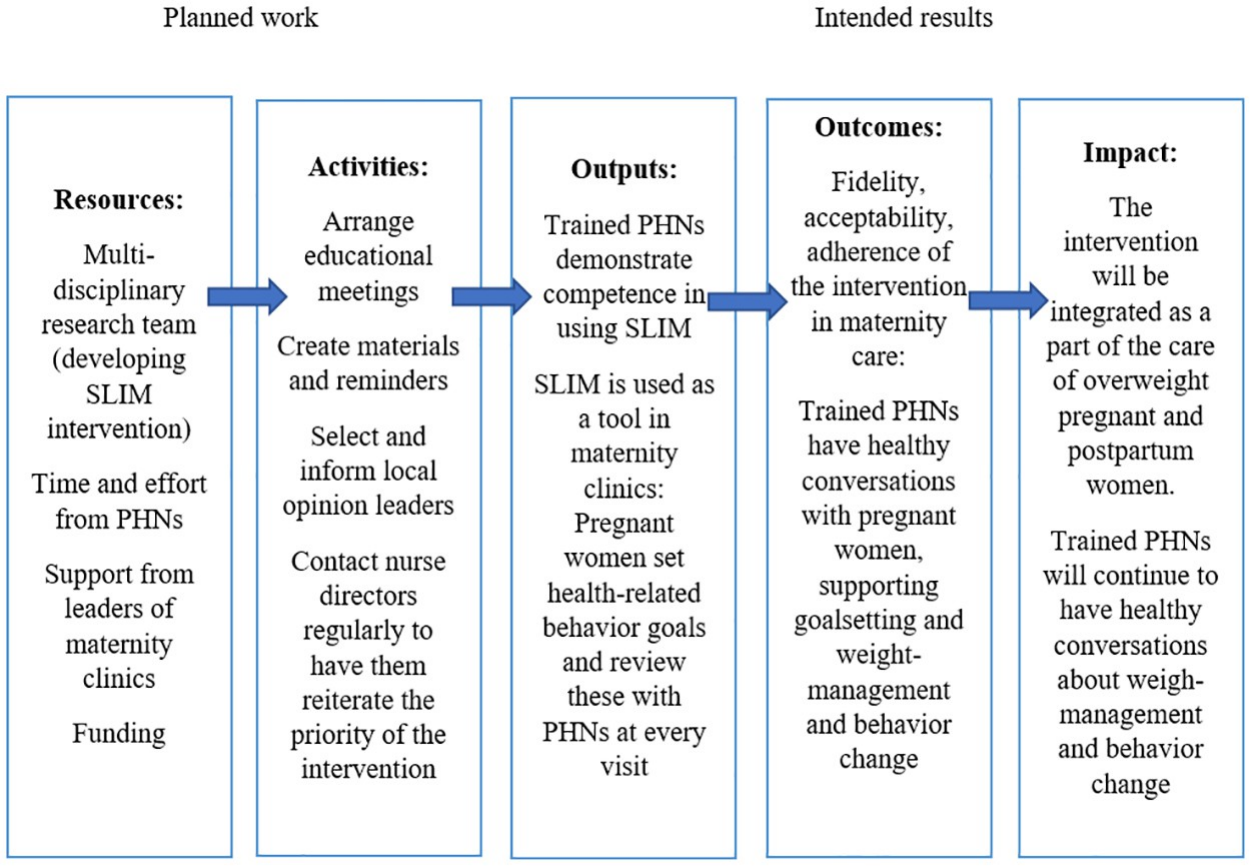
Logic model of implementation of SLIM intervention.

A process evaluation is being conducted during the intervention period using a mixed-methods approach to examine the feasibility and acceptability of the intervention, the implementation strategy and the study processes including the data collection [23]. To enable this evaluation, the components of the primary intervention (i.e., its intervention and delivery strategies), and the implementation outcomes (in terms of acceptability, fidelity, mechanism of impact and contextual influences) are being defined with the help of a logic model (Fig 3) [56, 57]. To evaluate the reach of the implementation, both the number of acceptances to participate and the number of refusals have been documented. In addition, the level of missing data in returned questionnaires is being assessed.

#### Data collection for process evaluation

*Public health nurses*. The PHNs perception of the appropriateness, acceptability and feasibility of the intervention is being evaluated with validated AIM, IAM & FIM questionnaires [58] every six months. In addition, all PHNs will be invited to take part in focus group exit interviews to assess the fidelity and adoption of the intervention at the end of the study. A semi-structured interview guide will be used and questions will aim to explore if the intervention was delivered as planned and whether adaptations to the intervention were made during delivery. Beliefs about the quality of the intervention delivery, the capacity and resources needed to deliver the intervention and the contextual influences on intervention implementation will also be explored. In addition, in order to assess the fidelity of the intervention, PHNs will complete a logbook of those contents of the participant’s maternity care appointments that are related to the SLIM intervention.

*Overweight women*. Women’s satisfaction with and usage of the SLIM intervention will be assessed using a validated questionnaire measuring appropriateness, acceptability and feasibility (AIM, IAM & FIM) of the intervention [58]. In addition, women will be asked to participate in a semi-structured phone interview at the end of the follow-up. A semi-structured interview guide will be used that will include questions aiming to capture the women’s perspectives on using the intervention. The daily wear time of the Oura-ring will be used to assess the fidelity of the intervention.

**The Acceptability of Intervention Measure (AIM), Intervention Appropriateness Measure (IAM), and Feasibility of Intervention Measure (FIM)** are four-item validated measures of implementation outcomes. Responses are measured on a scale from 1 to 5: 1 = Completely disagree, 2 = Disagree, 3 = Neither agree nor disagree, 4 = Agree, 5 = Completely agree. Scales can be created for each measure by averaging responses. The score is calculated by taking the sum of the four items. Higher scores indicate greater acceptability, appropriateness, or feasibility. Cronbach’s alphas should be 0.85 for acceptability, 0.91 for appropriateness, and 0.89 for feasibility using the 4-item scales from the structural validity survey, and 0.83 for acceptability, 0.87 for appropriateness, and 0.88 for feasibility using the test-retest reliability survey [58].

For those measures used in this study that did not have a validated translation, we undertook a translation from English to Finnish. Following the guidelines for translation and cultural adaptation of research questionnaires [59], the material was first translated by a professional translator to the target language. Next, the researchers (JS and HNV) made minor modifications to reflect the local cultural context, and this version was subsequently translated back to English by another independent translator. After back-translation, the accuracy of the translation was verified by the researchers (JS and HNV) and any discrepancies discussed until a consensus was reached. The AIM, IAM & FIM was translated in collaboration with its original developer, Prof. Bryan Weiner.

#### Data analysis of the process evaluation

Qualitative data, recorded interviews, will be transcribed verbatim and transferred to a NVivo Version 12.1.0 template. Data will be analyzed using thematic analysis [60]. Quantitative data will be analyzed statistically. Descriptive statistics will be calculated to AIM, IAM and FIM questionnaires and logbooks. Qualitative and quantitative data will be integrated as a part of the process evaluation. The change in AIM, IAM and FIM will be analyzed between pregnancy week 34 and 12 weeks after birth, using paired t-tests or Wilcoxon signed rank tests.

### Ethical considerations

The study is conducted in accordance with the Helsinki Declaration and approved by the Joint Ethics Committee of the Hospital District of Southwest Finland (113/1801/2020) and the maternity clinics. The trial is registered at the Clinicaltrials.gov register platform (ID NCT04826861). Written informed consent has been obtained from the participants.

### Trial status

Recruitment of participants to the SLIM study was initiated in April 2021 and the trial is progressing according to plan. This protocol was finalized before the research team received any data.

## Discussion

Postpartum weight retention increases women’s future cardiometabolic risks and obesity in subsequent pregnancies. In addition, the offspring of overweight or obese women have an increased risk of obstetric morbidity and mortality, as well as a long-term risk of childhood obesity and metabolic dysfunction. Nonetheless, half of overweight or obese women gain more weight than that recommended by the Institute of Medicine gestational weight guidelines [61]. Systematic reviews and meta-analysis show that gestational weight gain was associated with postpartum weight retention up to 21 years postpartum, suggesting that gestational weight gain outside the recommendations can lead to short-and long-term weight management challenges [62]. In contrast, increase in self-efficacy in weight management has been associated with dietary intake, physical activity levels and weight loss among sedentary overweight or obese adults [63].

The SLIM intervention was developed in collaboration with overweight women and PHNs working in maternity clinics with the aim of increasing self-efficacy in weight management. Developing feasible implementation strategies to support the provision of effective weight-management interventions is crucial and implementing complex interventions in different contexts usually requires tailoring. The process evaluation focuses on the implementation process and attempts to determine how successfully the project followed the strategy laid out in the logic model. In addition, the process evaluation focuses on the acceptability of the intervention and its evaluation and it also enables the researchers to distinguish adaptations that can be made in different contexts [57].

The strengths of this study can be found in the theory-based development of the trial and also in including the end users in the intervention and trial development. A potential limitation of this study is the selection bias in participant recruitment and retention; as weight is a sensitive subject and participation is voluntary, it is expected that those overweight women willing to participate are already motivated to make behavioral change, and this will probably result in more positive findings. There will most likely be a number of overweight women that would benefit from this intervention, but did not want to participate, e.g., women with mental problems that are often related to being overweight; these women would also be more challenging to motivate into behavioral change. Another limitation is our relatively long follow-up time, which might result in high drop-out rates. However, possible drop-outs were considered in the power calculations so that the results reached the necessary sample size.

The SLIM intervention has a strong potential to increase overweight pregnant women’s self-efficacy, limit excessive gestational weight gain and also prevent postpartum weight retention. This may impact the lifestyle and long-term health of these women as well as their families. Our long-term aim is to involve communal care maternal clinics in promoting women’s healthy lifestyle, improved self-efficacy, and healthy weight management.

## Supporting information

S1 Checklist SPIRIT 2013 checklist: Recommended items to address in a clinical trial protocol and related documents*. (DOC)

S1 Table Links between COM-B model, TDF domains, intervention functions and behavior change techniques (BCTv1) for the SLIM intervention. (DOCX)

S2 Table TIDieR, SLIM intervention for overweight pregnant women. (DOCX)

S3 Table Specifications of implementation strategies. (DOCX)

S1 File Study protocol_Finnish. (DOCX)

S2 File Study protocol_English. (DOCX)
