# Supplementary material for: Supporting lifestyle change in obese pregnant mothers through the wearable internet-of-things (SLIM) -intervention for overweight pregnant women: Study protocol for a quasi-experimental trial
Source: PLoS One. 2023 Jan 19;18(1):e0279696. doi: 10.1371/journal.pone.0279696 (PMC9851496; doi:10.1371/journal.pone.0279696)
Supplement: S1 Table — (DOCX) [file pone.0279696.s002.docx]

| **COM-B component** | ***TDF domain* linking to COM-B**  **components** | ***Intervention function:* examples of behaviour change techniques (BCTv1)** | **Examples of intervention strategies** |
| --- | --- | --- | --- |
| Physical capability | *Skills:*  Lack of skills needed to cook healthy food  Lack of skills needed to follow instructions for healthy lifestyle choices (Barriers) | *Education:* Information about safe exercise during pregnancy    *Training:* how to exercise, how to cook healthy food | An educational seminar  at the start of intervention  Written information  at different stages, food diary and Oura application.  Demonstration given by  researcher on how to use the applications    PHNs give information about health consequences of overweight and explain the benefits of regular circadian rhythm and healthy lifestyle in each antenatal visit  Overweight women set nutritional and/or physical activity and/or sleep hygiene goals in collaboration with public health nurses  Overweight women set weight gain goals if possible  Health technology: Overweight women are given Oura rings and electronic food diary. They are asked to self-monitor their behavior.  Health technology provides data to revising goals  Partners are supported to participate antenatal visits and healthy lifestyle (Family centered counseling)  The PHNs check the data to evaluate PA, sleep and nutrition. PHNs support and create goals in collaboration with the overweight women in each antenatal visit  The PHNs give feedback using motivational interviewing and solution centered counseling |
| Psychological capability | *Knowledge:*  Lack of professional information about the risks of obesity  Lack of concrete counseling on how to eat or exercise  Lack of consistent ways to broach the weight management topic  (Barriers)  *Memory, attention and decision processes:*  Overweight as a part of women’s identity (Barrier)  Individual weight management counseling (Facilitator)  Previous experiences with weight management (Facilitator or barrier)  *Behavioral regulation:*  Utilizing information from health technology (smart wearables) in antenatal visits (Facilitator) | *Education:* Information about health consequences; feedback  on behaviour; feedback on outcome(s) of behaviour; prompts/cues; self-monitoring  of behaviour  *Persuasion:* Adding objects to the environment  *Enablement:* Goal setting (behaviour);  goal setting (outcome); self-monitoring of behaviour; action planning    *Training:*  Demonstration of the  behaviour; instruction on how to perform a behaviour; self-monitoring of behaviour;  review behavioural goals |  |
| Physical opportunity | *Environmental context and resources:*  Pregnancy related physical discomfort  Lack of time during antenatal visits  Lack of postnatal counseling (Barriers) | *Environmental Restructuring*  *and Enablement:* Restructuring  the physical environment.  Adding objects to the  environment, self-monitoring of behavior |  |
| Social opportunity | *Social influences:*  Support from partners and family  Peer support (Facilitators) | *Enablement:* Social support |  |
| Automatic motivation | *Reinforcement:*  Motivating counseling  Positive feedback  Encouragement to make small changes  Asking questions and listening (Facilitators)  Tone of voice  (Facilitator or barrier)    *Emotions:*  Despair and feelings of shame  Denial and defense reactions  Low self-esteem  Psychological reasons for eating (Barriers)  Discreet counseling (Facilitator) | *Environmental Restructuring:*  Prompts/ cues via phone app    *Persuasion:* Self-monitoring  of behaviour; feedback on behavior, review behavioral goals    *Training:* self-reward; habit formation |  |
| Reflective motivation | *Social/professional role and identity:*  Conflict: Public health nurses’ self-described role as advisors, which conflicted with overweight women’s expectation of a tough stance and professional counseling  Overweight women’s reluctance to take responsibility for their actions (Barriers)    *Beliefs about capability:*  Difficult to change lifestyle  Lack of desire to change their lifestyle on the part of some women (Barriers)  Mapping the women’s life situations (Facilitator)    *Beliefs about consequences:*  Pregnancy as an excuse (Barriers)  Unborn child as a source of motivation  (Facilitators)    *Optimism:*  Women’s belief that they could exercise after birth just as they did before pregnancy (Facilitator)  *Intentions:*  Willingness to change but a lack of motivation to make concrete changes (Barrier)  Intentions to change lifestyle after the baby’s birth (Facilitator)    *Goals:*  Paternalistic goals made by public health nurses (Barrier)  Goals made in collaboration with public health nurses (Facilitator) | *Persuasion:* Information about consequences; feedback on behaviour; feedback on outcome(s) of behaviour;    *Environmental restructuring:*  Adding objects to the environment; Self-monitoring of behaviour;  Goal setting (behaviour)    *Enablement:* feedback via phone app,  feedback via maternity care; goal setting    *Education:* Information about health consequences |  |
