# Supplementary material for: Supporting lifestyle change in obese pregnant mothers through the wearable internet-of-things (SLIM) -intervention for overweight pregnant women: Study protocol for a quasi-experimental trial
Source: PLoS One. 2023 Jan 19;18(1):e0279696. doi: 10.1371/journal.pone.0279696 (PMC9851496; doi:10.1371/journal.pone.0279696)
Supplement: S2 Table — (DOCX) [file pone.0279696.s003.docx]

| *SLIM intervention* | |
| --- | --- |
| *Why?* | Maternal overweight, defined as a body mass index (BMI) of 25 or higher is a global public health concern (Ng et al 2014). In Finland 41.7% of pregnant women were overweight and 17.6 % obese; these numbers are rising over time (THL 2021). Maternal overweight increases risks for both, mother, and her child; for example, gestational diabetes, caesarean sections, preterm birth and asphyxia-related complications at birth (European Perinatal Health Report 2015; THL 2020; Shrestha et al 2021). Although previous research reported that weight-management interventions reduce weight gain during pregnancy. (i-WIP 2017), a gap exists between research evidence and practice in maternity care. The implementation of weight management interventions is challenging for several reasons, for example, obesity is often difficult to discuss with pregnant women and interventions may not have a major instant effect. |
| *What?* | ·       The intervention was developed based on the findings from interviews with overweight women and their care givers and during workshops in collaboration with PHNs working in maternity clinics.  ·       PHNs in maternity clinics provide support with special attention to promoting welfare and health, including guidance and support as regards sleep, physical activity and nutrition and food recommendations for pregnant and breastfeeding mothers (Hakulinen et al 2022). They also provide weight gain recommendations based on pre- pregnancy BMI and guidance for weight management; this is to encourage pregnant and postpartum women´s willingness to change and become motivativated to take care of their own and their unborn child 's health. The recommendations are based on the current evidence-based clinical practice care guidelines in Finland. (Lihavuus (lapset, nuoret ja aikuiset): Käypä hoito suositus, 2021).  ·       SLIM intervention includes four core components. The maternity care support and guidance used in the SLIM intervention is implemented using health technology as a part of normal maternity care visits. PHN check the data of 1) health technology, including Oura smart ring and the food diary to evaluate PA, sleep and nutrition. They then 2) give feedback using 3) motivational interviewing and 4) create goals in collaboration with the women participants. The goals are document in the patient’s records. |
| *Who?* | Overweight pregnant women and PHNs |
| *How?* | ·       1st antenatal visit: PHN introduces the SLIM intervention and ask permission for the researchers to contact the new participant.  ·   A researcher meets the participant: Instructions given for the Oura- smart ring and the application.  ·   Continuous monitoring of health parameters until 12 weeks after birth. Utilization of data from application as a part of weight-management counselling. |
| *Where?* | In maternity clinics reception room |
| *When and how much?* | - After recruitment participants start wearing Oura-smart rings continuously until 12 weeks after delivery.   - At every antenatal visit: PHN will check the data in their PC or participants mobile device to evaluate PA, sleep and nutrition. The PHNs will give feedback using motivational interviewing and solution centered counseling. They will create goals in collaboration with the participant and document it in their patient records. - A researcher will visit the maternity clinics every 6 months (interviews/workshops with PHNs) |
| *Tailoring* | The intervention will be conducted in a similar manner with every participant. Goals will be set individually based on each woman´s capability, opportunity and motivation. |
| *Modifications* | Usability and fidelity of the intervention will be evaluated in workshops with PHNs and modifications will be discussed if necessary. |
| *How well?* | The feasibility, acceptability, fidelity and receipt of the intervention will be assessed during and after the follow-up time from the perspectives of both PHNs (questionnaires, workshops, interviews) and overweight participants (questionnaires, health parameters, interviews) |
