# Supplementary material for: Supporting lifestyle change in obese pregnant mothers through the wearable internet-of-things (SLIM) -intervention for overweight pregnant women: Study protocol for a quasi-experimental trial
Source: PLoS One. 2023 Jan 19;18(1):e0279696. doi: 10.1371/journal.pone.0279696 (PMC9851496; doi:10.1371/journal.pone.0279696)
Supplement: S3 Table — (DOCX) [file pone.0279696.s004.docx]

|  | Educational meetings /workshops | Educational materials | Local opinion leaders | Mandate change | Reminders |
| --- | --- | --- | --- | --- | --- |
| *Actor* | Researchers | Researchers | Selected nurses working in maternity clinics | Director nurses in maternity clinics | Researchers |
| *Action* | Provides oral information about intervention and implementation | Provides written information about intervention and implementation | Supports implementation process and collaborate with research team members regularly. | Leadership declares the importance of the intervention and their determination to have it implemented | Send reminders via email |
| *Target of the action* | Nurses working in maternity clinics | Nurses working in maternity clinics | Nurses working in maternity clinics | Nurses working in maternity clinics | Nurses working in maternity clinics |
| *Temporality* | Before recruitment begins, until 1 years | Before recruitment begins | During the whole follow-up period | During the whole follow-up period | During the whole follow-up period |
| *Dose* | In every 6 months | Once | Monthly | In every 1 or 2 months | In every 3 months |
| *Implementation outcome affected* | *Acceptability:* Nurses experiences pre-implementation  *Fidelity:* Nurses involvement.  *Adoption:*  “uptake” of intervention. | *Fidelity;* adherence to the program.  *Adoption;* “uptake” of intervention. | *Acceptability:* Nurses experiences throughout the implementation | *Fidelity:* adherence to the intervention.  *Adoption:* “uptake” of intervention.  *Acceptability:* Nurses experiences pre- and throughout the implementation | *Fidelity:* adherence to the intervention.  *Adoption:*  “uptake” of the intervention. |
| *Justification* | Education - BCW | Education - BCW | Training /enablement – BCW | Persuasion / coercion / Incentivisation / environmental restructuring - BCW | Persuasion / coercion /Incentivisation - BCW |
