# Supplementary material for: Supporting lifestyle change in obese pregnant mothers through the wearable internet-of-things (SLIM) -intervention for overweight pregnant women: Study protocol for a quasi-experimental trial
Source: PLoS One. 2023 Jan 19;18(1):e0279696. doi: 10.1371/journal.pone.0279696 (PMC9851496; doi:10.1371/journal.pone.0279696)
Supplement: S1 File — (DOCX) [file pone.0279696.s005.docx]

**Terveysteknologiaa hyödyntävän painonhallintaintervention muodostaminen ja käyttöönottoprosessin kehittäminen ylipainoisille odottajille**

*Johanna Saarikko, TtM, tohtorikoulutettava, Hoitotieteen laitos, Turun yliopisto*

*Hannakaisa Niela-Vilén, TtT, erikoistutkija, Hoitotieteen laitos, Turun yliopisto*

*Emilia Huvinen, LT, HUS Akuutti, Teratologinen tietopalvelu, Helsingin yliopisto ja Helsingin yliopistollinen Sairaala*

*Eeva Ekholm, LT, dosentti, Synnytys- ja naistentaudit, Turun yliopistollinen keskussairaala*

*Iman Azimi, FT, Tulevaisuuden teknologioiden laitos, Turun yliopisto*

*Fatemeh Sarhaddi, tohtorikoulutettava, Tulevaisuuden teknologioiden laitos, Turun yliopisto*

*Milad Asgari, tohtorikoulutettava, Computer science, University of California, Irvine, USA*

*Amir Rahmani, TkT, MBA, dosentti, University of California, Irvine, USA,*

*Pasi Liljeberg, FT, professori, Tulevaisuuden teknologioiden laitos, Turun yliopisto*

*Anna Axelin, TtT, apulaisprofessori, Hoitotieteen laitos, Turun yliopisto*

1. **Tutkimuksen tausta**

Lihavuus (BMI ≥ 30) on maailmanlaajuisesti kasvava ongelma. Teollisissa maissa lihavuus on yksi merkittävimmistä kuolinsyistä ja taudinaiheuttajista (WHO 2018). Odottajien keskuudessa lihavuus lisääntyy niin ikään ja on merkittävä huolenaihe julkisessa terveydenhuollossa (Ng ym. 2013). Suomessa 36 % odottajista on ylipainoisia (BMI ≥ 25) ja 14 % lihavia, (THL 2017). Äidin painolla ennen raskautta sekä sen aikana on pitkäaikaisia vaikutuksia myös syntyvän lapsen terveyteen. Raskautta edeltävä ylipaino lisää riskiä esimerkiksi sikiön makrosomiaan, synnytyskomplikaatioihin, synnytyksen aikaisiin toimenpiteisiin ja sektioihin (European Perinatal Health Report 2015). Lihavilla odottajilla on myös suurempi riski sairastua raskauden aikaiseen diabetekseen ja pre-eklampsiaan (Pallasmaa ym. 2015). Lisäksi lihavien naisten lapset tulevat todennäköisemmin olemaan ylipainoisia tai lihavia (Poston 2012).

Liiallisen raskaudenaikaisen painonnousun ennaltaehkäisy on tärkeää erityisesti naisille, jotka ovat ylipainoisia jo ennen raskautta. Raskausajan painonhallintainterventiot vähentävät painonnousua raskauden aikana (i-WIP 2017). Raskausajan painonhallintainterventiot vähentävät myös synnytyksen jälkeistä masennusta sekä painon kertymää, joten interventioilla on mahdollista saavuttaa myös merkittäviä pitkäaikaisvaikutuksia sekä äidin että lapsen terveydentilalle (Brown ym. 2017). Ylipainoisia äitejä tulisi myös erityisesti kannustaa imetykseen, jonka on todettu vähentävän diabeteksen sekä sydän- ja verisuonitautien riskiä ja keskivartalolihavuutta. Lisäksi imettävillä naisilla on todettu vähäisempää raskauksien kerryttämää ylipainoa sekä pienempää riskiä lihavuudelle myöhemmin elinkaaren aikana. (Dutton ym. 2018; Bobrow ym. 2013.)

Vaikka painonhallintainterventioiden vaikuttavuus on osoitettu, interventioiden käyttöönotto käytännössä on haastavaa. Implementaatiotutkimukselle on siten ilmeinen tarve. Ensimmäisessä osatutkimuksessamme (raportointivaiheessa) kuvattiin laadullisella tutkimusasetelmalla terveydenhuollon ammattilaisten sekä odottajien ja synnyttäneiden naisten kokemuksia ylipainoisten odottajien painonhallinnanhoitokäytännöistä. Käyttäytymisen muutospyörää (Behaviour of Change Wheel, BCW) hyödyntäen aineistosta tunnistettiin painonhallintaintervention keskeiset vaikuttamistoiminnot (intervention functions), joita olivat *koulutus, suostuttelu, mahdollistaminen, ympäristön muuttaminen ja harjoittelu.* BCW-teorian mukaisesti muodostettiin käyttäytymisen muutostekniikat (Behaviour change techniques taxonomy v1): *käyttäytymistavoitteiden asettaminen, lopputulos tavoitteiden asettaminen, käyttäytymistavoitteiden uudelleen arviointi, käyttäytymisen omaseuranta, keinojen lisääminen ympäristöön, tietoa terveysvaikutuksista ja palaute käyttäytymisestä* (Michie ym 2011) ja implementaatiostrategiat, jotka luokiteltiin Expert Recommendations for Implementing Change (ERIC) -kompilaation (Powell ym. 2015) ja Proctor ym (2013) suosituksia mukaillen.

Terveysteknologiaa voidaan hyödyntää intervention toteuttamisessa käyttöönotossa. Aiemmassa käytettävyystutkimuksessamme odottajat suhtautuivat myönteisesti rannekkeeseen, jolla mitattiin odottajien aktiivisuutta, unta ja sykettä. Seitsemän kuukauden seuranta-ajasta odottajat käyttivät aktiivisuusranneketta keskimäärin hieman yli 5 kuukautta (Saarikko ym. 2020; Grym ym. 2019). Etämonitorointisysteemillä olisi mahdollista seurata jatkuvasti odottajien fysiologisia parametreja, aktiivisuutta ja ravitsemusta osana innovatiivista ja painonhallintainterventiota. Lisäksi etämonitoroinnin avulla voidaan todennäköisesti tukea intervention implementaatiota.

1. **Tutkimuksen tavoite ja tutkimustehtävät**

Tutkimuksen tarkoituksena on testata ensimmäisessä osatutkimuksessa kehitetyn näyttöön perustuvan, terveysteknologiaa hyödyntävän painonhallintaintervention vaikuttavuutta ja käyttöönoton onnistumista ylipainoisten odottajien painonhallintaan äitiysneuvoloissa. Tutkimuksen tavoitteena on kehittää interventiota ja käyttöönottoprosessia tarkastelemalla toteutuksen tason ja intervention vaikuttavuuden välistä yhteyttä.

***Tutkimuskysymykset:***

1. Kuinka vaikuttava interventio on minäpystyvyyden lisäämisessä raskauden aikana ja synnytyksen jälkeen?
2. Kuinka vaikuttava interventio on painonhallinnassa, masennusoireiden lievittämisessä sekä elämänlaadun ja elämänhallinnan parantamisessa raskauden aikana ja synnytyksen jälkeen?
3. Kuinka hyvin intervention toteutus vastasi suunnittelua?
4. Kuinka hyvin interventio soveltuu ylipainoisille odottajille käytettäväksi äitiysneuvoloissa?

***Tutkimushypoteesi:*** Ylipainoisten odottajien minäpystyvyys paranee raskauden edetessä ja synnytyksen jälkeen.

**3. Tutkimuksen empiirinen toteutus**

Tutkimus keskittyy intervention vaikuttavuuden ja käyttöönoton arviointiin mixed-method tutkimusmenetelmän avulla ennen-jälkeen tutkimusasetelmalla. Tutkimukseen rekrytoidaan 1.4.2021–31.5.2022 sekä ylipainoisia odottajia että äitiysneuvoloiden terveydenhoitajia. Ylipainoiset odottajat rekrytoidaan VSSHP:n kuntayhtymän äitiysneuvoloista terveydenhoitajien välityksellä ensimmäisen neuvolakäynnin yhteydessä. Mukaanottokriteereinä ovat 1) täysi-ikäisyys, 2) suomenkielisyys, 3) ylipaino (BMI > 25) 4) raskaus < 15 viikkoa ja 5) kykenevyys antaa tietoon perustuva suostumus. Tutkimuksesta suljetaan pois odottajat, 1) jotka eivät omista älylaitetta, johon tutkimuksessa käytettävät sovellukset on mahdollista ladata, 2) joilla on diagnosoitu vaikea psyykkinen sairaus (esim. skitsofrenia), 3) joilla on ennen raskautta diagnosoitu insuliinihoitoinen diabetes tai 4) fyysinen liikuntarajoite. Otoskoko (n = 54) laskettiin minäpystyvyydessä odotettavan muutoksen (Clarke ym. 1996) perusteella: efektikoko d = 0,8, voima β = 90 % ja luottamusväli 95 % (yhden otoksen, yksisuuntainen t-testi). Kadoksi arvioitiin 20 %. Osallistujia informoidaan tutkimuksesta (liitteet 4a ja16c) ja he täyttävät suostumuslomakkeen (liite 5a). Osallistujille annetaan käyttöön Oura-älysormus ja he lataavat SLIM-sovelluksen älypuhelimeensa. Äitiysneuvoloiden terveydenhoitajat (n = 15) on rekrytoitu jo tutkimuksen ensimmäisessä vaiheessa. Mukaanottokriteerinä on työskentely äitiysneuvolassa.

**4. Interventio**

*Intervention* tarkoituksena on parantaa ylipainoisten odottajien minäpystyvyyttä. Interventio toteutetaan osana neuvolan ohjausta alkaen ensimmäisestä neuvolakäynnistä jatkuen jokaisella äitiysneuvolakäynnillä kolme kuukautta synnytyksen jälkeen. Intervention keskeiset komponentit ovat: *tavoitteiden asettaminen, motivoiva haastattelu, palaute* ja *terveysteknologia,* johon sisältyy Oura-sormus, sähköinen ruokapäiväkirja sekä SLIM-sovellus. Oura on kevyt, vedenpitävä älysormus, jonka avulla on mahdollista mitata esimerkiksi sykettä ja sykevaihtelua, askeleita, aktiivisuustasoja, unta ja kehon lämpöä. Ouraa on mahdollista käyttää kummassa kädessä tahansa. SLIM-sovelluksen kautta lähetetään sähköiset kyselylomakkeet ja mittarit sekä muistutuksia vastaamisesta. Sovellukseen linkitetään myös Ouran ja sähköisen ruokapäiväkirjan data. Terveydenhoitajat hyödyntävät Oura-sovelluksen, ruokapäiväkirjan ja TFEQ-R18 ja 6-FQ mittareiden tietoja neuvolakäynneillä. Oura-sovelluksen ja ruokapäiväkirjan dataa tarkastellaan yhdessä odottajan kanssa. Motivoivaa haastattelua hyödyntämällä annetaan palautetta ja asetetaan tavoitteet, jotka kirjataan potilastietojärjestelmään. Interventio on kuvattu tarkemmin liitteessä 16a sekä kuviossa 1.

Terveydenhoitajat kutsutaan koulutuspalaveriin ennen rekrytoinnin aloitusta. Tutkija esittelee palaverissa intervention ja sen toteutukseen liittyvät asiat, sekä koulutusmateriaalit (liite 12j). Terveydenhoitajat saavat lisäksi tutkimustiedotteen (liite 4b) ja heiltä pyydetään tietoon perustuva suostumus (liite 5b). Tämän jälkeen järjestetään tutkimustapaamisia kuuden kuukauden välein workshopien ja haastattelujen muodossa. Ensimmäisen palaverin yhteydessä valitaan yksikön sisältä paikallinen vaikuttaja, joka toimii jatkossa tärkeässä roolissa tukihenkilönä muille ja yhteyshenkilönä tutkijoihin. Johtavia hoitajia pyydetään kuukausipalaverien yhteydessä ottamaan interventio puheeksi ja siten kannustamaan terveydenhoitajia intervention jatkuvaan toteutukseen. Implementointistrategiat on kuvattu tarkemmin liitteessä 16 b.

Kohderyhmä 1: n=54 ylipainoista odottajaa

Rekrytointi ensimmäisellä neuvolakäynnillä: Oura-älysormus, FatSecret ja SLIM-sovellus

SLIM:in kautta: Lähtötilanteen kartoitus (taustatietolomake, WEL, PASE, EPDS, TFEQ-R18, 6-FQ, WHOQOL-BREF, Koettu stressi -kysely ruokapäiväkirja)

Jokaisella neuvolakäynnillä: Ouran datan perusteella: palaute, motivoiva haastattelu, tavoitteiden asettamien ja kirjaus. Viikoittaiset muistutukset SLIM:in kautta.

Raskausviikko 20:

AIM, IAM & FIM-mittarit SLIM:in kautta

Raskausviikko 34: WEL, PASE, EPDS, TFEQ-R18, 6-FQ, WHOQOL-BREF, Koettu stressi -kysely, ruokapäiväkirja. AIM, IAM & FIM -mittarit

2 kk synnytyksen jälkeen:

TFEQ-R18, 6-FQ-kyselyt, AIM, IAM & FIM-mittarit SLIM:in kautta.

3 kk synnytyksen jälkeen

Ouran datan perusteella: palaute, motivoiva haastattelu, tavoitteiden asettamien ja kirjaus. WEL, PASE, EPDS, TFEQ-R18, 6-FQ, WHOQOL-BREF, Koettu stressi -kyselyt, loppuhaastattelu.

Kohderyhmä 2: äitiysneuvoloiden työntekijät (n=15)

AIM, IAM & FIM -mittarit 6 kk välein

Haastattelu / workshop:

6 kk välein

**Aineistonkeruu: 1.4.2021– 31.8.2022**

*Kuvio 1. Intervention kuvaus ja toteutusaikataulu.*

**5. Tutkimuksen aineistonkeruumenetelmät**

***Vaikuttavuus***

Tutkimuksen ensisijainen vastemuuttuja on odottajan minäpystyvyys. Muita vastemuuttujia ovat odottajan paino, masennusoireet, elämänhallinnantunne ja elämänlaatu. Aineistonkeruumenetelminä käytetään ylipainoisten odottajien osalta älysormusta, sähköistä ruokapäiväkirjaa sekä SLIM-aplikaatiota. SLIM aplikaation kautta lähetetään taustatietolomake (liite 12a) ja validoidut mittarit: Weight Efficacy Life-Style Questionnaire (WEL) (Clark ym 1991)(liite 12b), Self-Efficacy for Physical Activity Scale (PASE) (Marcus ym 1992) (liite 12c), 6-FQ (Kushner ym 2016) (liite 12d), TFEQ-R18 (Karlsson ym 2000) (liite 12e), WHOQOL-BREF (WHOQOL group 1998) (liite 12f), SOC-13 (Antonovsky 1987) (liite 12g), Koettu stressi (Huizink 2016 & Cohen ym 1983)(liite 12h) ja EPDS (Cox ym 1987)(liite 12i). Lisäksi aineistoa kerätään potilastietojärjestelmistä sekä puhelinhaastatteluiden avulla (liite 16f).

***Käyttöönoton arviointi***

Intervention toteutuksen tasoa tarkastellaan prosessievaluaation mukaisesti kontekstiin, implementointiin ja vaikutusmekanismeihin liittyen (taulukko 1) (Moore ym. 2015). Aineistonkeruumenetelminä käytetään terveydenhoitajien osalta haastatteluita ja workshopeja (n = 3), joiden avulla arvioidaan kontekstiin liittyviä asioita, sekä intervention heikkoja tai keskeneräisiä kohtia. Haastattelurunko on kuvattu liitteessä x. Intervention toteutususkollisuutta (fidelity) arvioidaan seurantalomakkeiden avulla (liite 12l) ja intervention käyttöä (dose) potilasjärjestelmien kirjausten perusteella. Lisäksi tutkitaan intervention hyväksyttävyyttä (acceptability), soveltuvuutta (appropriateness) ja toteutettavuutta (feasibility) AIM, IAM & FIM -mittareiden (Weiner ym 2017) avulla, jotka lähetetään terveydenhoitajille sähköpostitse kuuden kuukauden välein ja odottajille 20 ja 34 raskausviikoilla sekä 2 kuukautta synnytyksen jälkeen. Tutkimuksesta kieltäytyvien määrä ja spontaanisti ilmoitetut syyt kieltäytymiselle kirjataan ylös, samoin tutkimuksen keskeyttäneiden määrä ja mahdollisesti annetut syyt. Näiden perusteella arvioidaan, kuinka hyvin kohderyhmä saavutettiin (reach).

*Taulukko 1 Prosessievaluaatio: intervention toteutuksen arviointi*

| Prosessievaluaation komponentti | Komponentin arviointi | Lähdeaineisto |
| --- | --- | --- |
| Implementointi | Intervention ja implementaatiostrategioiden   - Toteutususkollisuus (fidelity) - Intervention käyttö (dose) - Intervention saavutettavuus (reach) | - Seurantalomake terveydenhoitajille - Potilastietojärjestelmät - Tutkimuksen kieltäytymis- ja keskeytysluvut |
| Vaikutusmekenismi | Terveydenhoitajien ja ylipainoisten odottajien suhtautuminen interventioon ja sen toteutukseen   - Toteututettavuus (feasibility) - Hyväksyttävyys (acceptability) - Soveltuvuus (appropriateness) | - Kyselyt odottajille: (AIM, IAM, FIM) - Kyselyt terveydenhoitajille: (AIM, IAM, FIM) - Terveydenhoitajien haastattelut ja workshopit |
| Konteksti | - Intervention toteutusta edistävät ja estävät tekijät - Implementaatiostrategioiden toteutusta edistävät ja estävät tekijät | - Terveydenhoitajien haastattelut ja workshopit |

Aineiston analyysi toteutetaan SLIM-sovelluksesta saatavan datan ja kyselylomakkeiden osalta tilastollisin menetelmin yhden otoksen yksisuuntaisella t-testillä. Haastattelut analysoidaan litteroimalla tallennettu haastatteluaineisto ja analysoimalla se aineistolähtöisen sisällönanalyysin avulla. Raportoitujen kieltäytymis- ja keskeyttämisprosenttien ja annettujen syiden perusteella parannetaan interventioon sitoutumista poistamalla interventiosta osallistumista estäviä tekijöitä tai parantamalla sen käytettävyyttä. (Grol ym. 2013; Moore ym 2015.)

***Tutkimusluvat ja resursointi***

Tutkimukselle haetaan lausunto VSSHP:n eettiseltä toimikunnalta ja tutkimusluvat kuntayhtymien organisaatioista, jotka osallistuvat tutkimukseen.

**6. Tutkimuksen eettiset kysymykset**

Tutkimuksessa tullaan noudattamaan lakia (488/99) ja asetusta (986/99) lääketieteellisestä tutkimuksesta sekä lakia potilaan asemasta ja oikeuksista (785/92). Henkilötietojen siirto EU:n ja ETA:n ulkopuolelle perustuu komission hyväksymiin vakiolausekkeihin (standard contractual clauses, SCC) rekisterin pitäjän (Turun yliopisto) ja henkilötietojen käsittelijän (UCI) välillä. Koko tutkimuksen ajan noudatetaan hyvää tieteellistä käytäntöä Tutkimuseettisen neuvottelukunnan suositusten mukaan (TENK 2012). Henkilötietojen käsittelyn lisäsuojatoimenpiteenä on tehty vaikutustenarviointi (liite 16 d). Tutkittaville annetaan tietoa suullisesti ja kirjallisesti sekä muistutetaan osallistumisen vapaaehtoisuudesta. Tutkittavilta pyydetään kirjalliset tietoiset suostumukset tutkimukseen osallistumisesta. Raskaana olevat ovat tutkittavina erityisen haavoittuvia, mutta raskaana olevien riskiryhmien tutkiminen on tässä tutkimuksessa perusteltua, koska vastaavia tuloksia ei voida saada muilla tutkittavilla. Tutkimus ei aiheuta osallistujalle kajoavia toimenpiteitä eikä vaadi ylimääräisiä neuvola- tai sairaalakäyntejä eikä aiheuta kustannuksia. Tutkimukseen osallistuvilta pyydetään kirjallinen tietoon perustuva suostumus tutkimukseen osallistumisesta. Tutkimukseen rekrytoitavat saavat myös tarvittaessa aikaa harkita suostumustaan. Liitteessä 3 on kuvattu tarkemmin tutkimuseettisiä asioita.

Kerätyt tiedot käsitellään luottamuksellisesti, eikä niitä anneta ulkopuolisten käyttöön. Kerätty tutkimusaineisto ja sen käsittelyyn tarvittavat tietokoneet säilytetään asianmukaisesti lukituissa tiloissa. Terveysteknologian avulla kerätty aineisto käsitellään pseudonymisoituna. Turun yliopisto vastaa tutkimuksen yhteydessä tapahtuvan henkilötietojen käsittelyn lainmukaisuudesta. Tutkimusaineisto hävitetään viisi vuotta tulosten julkaisun jälkeen. Tietosuojaseloste liitteenä 16c. Kerätyn aineiston avulla on mahdollista saada tietoa painonhallintaintervention implementointiin vaikuttavista tekijöistä. Tietoja hyödynnetään jatkossa intervention laajemmassa käyttöönotossa. Tutkimuksesta saadut hyödyt ylittävät selvästi siitä koituvat haitat.

**Lähteet**

Antonovsky, A. 1987. Unraveling the mystery of health. How people manage stress and stay well. San Francisco: Jossey-Bass Publishers

Asetus lääketieteellisestä tutkimuksesta 986/1999. <https://www.finlex.fi/fi/laki/alkup/1999/19990986>

Brown J., Alwan N.A., West J., Brown S., McKinlay C.J.D., Farrar D., Crowther C.A. 2017. Lifestyle interventions for the treatment of women with gestational diabetes (Review). Cochrane Database Syst Rev. 4(5). CD011970. doi: 10.1002/14651858.CD011970.pub2.

Bobrow KL, Quigley MA, Green J, Reeves GK, Beral V for the Million Women Study Collaborators. 2013. Persistent effects of women’s parity and breastfeeding patterns on their body mass index: results from the Million Women Study. Int J Obes (Lond). 37(5):712-7. doi: 10.1038/ijo.2012.76.

Clark, M. M., Abrams, D. B., Niaura, R. S., Eaton, C. A., & Rossi, J. S. (1991). Self-efficacy in weight management. Journal of Consulting and Clinical Psychology, 59(5), 739–744. <https://doi.org/10.1037/0022-006X.59.5.739>

Clark, M.M., Cargill, B.R., Medeiros, M.L., Pera, V. 1996. Changes in Self-Efficacy Following Obesity Treatment.Obesity research 1996 ; 2(4) : 179-181.

Cohen S, Kamarck T, Mermelstein R. .1983 A global measure of perceived stress. Journal of Health and Social Behavior Vol. 24, No. 4, pp. 385-396

Cox, J.L., Holden, J.M., and Sagovsky, R. 1987. Detection of postnatal depression: Development of the 10-item Edinburgh Postnatal Depression Scale. British Journal of Psychiatry 150:782–6.

Dutton H, Borengasser SJ, Gaudet LM, Barbour LA, Keely EJ. 2018. Obesity in Pregnancy: Optimizing Outcomes for Mom and Baby. Med Clin North Am. 102(1), 87-106.

European commission 5.2.2010. Standard Contractual Clauses (SCC) Standard contractual clauses for data transfers between EU and non-EUcountries.

<https://ec.europa.eu/info/law/law-topic/data-protection/international-dimension-data-protection/standard-contractual-clauses-scc_en>

European Perinatal Health Report 2015. Core indicators of the health and care of pregnant women and babies in Europe in 2015. November 2018. <https://www.europeristat.com/images/EPHR2015_web_hyperlinked_Euro-Peristat.pdf> (21.12.2020).

Grol R., Wensing M., Eccles M., Davis D. (toim.) 2013. Improving Patient Care. The implementation of change in health care. Second edition. UK: Wiley Blackwell.

Grym K, Niela-Vilén H, Ekholm E, Hamari L, Azimi I, Rahmani A, et al. Feasibility of smart wristbands for continuous monitoring during pregnancy and one month after birth. BMC Pregnancy Childbirth 2019 Jan 17;19(1):34 [FREE Full text] [doi: 10.1186/s12884-019-2187-9] [Medline: 30654747]

Huizink AC, Delforterie MJ, Scheinin NM, Tolvanen M, Karlsson L, Karlsson H. Adaption of pregnancy anxiety questionnaire-revised for all pregnant women regardless of parity: PRAQ-R2. Arch Womens Ment Health. 2016;19(1):125-132. doi:10.1007/s00737-015-0531-2

i-WIP (The International Weight Management in Pregnancy) Collaborative Group. 2017. Effect of diet and physical activity based interventions in pregnancy on gestational weight gain and pregnancy outcomes: meta-analysis of individual participant data from randomised trials. BMJ 19(358) 3119. doi: 10.1136/bmj.j3119

Johansson S, Villamor E, Altman M, Bonamy AK, Granath F, Cnattingius S, 2014. Maternal overweight and obesity in early pregnancy and risk of infant mortality: a population based cohort study in Sweden. BMJ 2; 349: g6572. doi: 10.1136/bmj.g6572.

Karlsson J, Persson L-O, Sjöström L, Sullivan M. Psychometric properties and factor structure of the Three-Factor Eating Questionnaire (TFEQ) in obese men and women. Results from the Swedish Obese Subjects (SOS) study. Int J Obes 2000;12:1715-1725.

Kushner RF, Choi SW, Burns JL. Development of a six-factor questionnaire for use in weight management counseling. Patient Educ Couns 2016;99:2018-2025

Laki lääketieteellisestä tutkimuksesta 9.4.1999/488. <https://www.finlex.fi/fi/laki/ajantasa/1999/19990488>

Laki potilaan asemasta ja oikeuksista 17.8.1992/785. <https://www.finlex.fi/fi/laki/ajantasa/1992/19920785>

Marcus BH, Selby VC, Niaura RS, Rossi JS. Self-efficacy and the stages of exercise behavior-change. Res Q Exerc Sport. 1992;63:60–6.

Michie S, van Stralen MM, West R. 2011. The behaviour change wheel: A new method for characterising and designing behaviour change interventions. Implement Sci. 23; 6:42. doi: 10.1186/1748-5908-6-42.

Moore GF, Audrey S, Barker M, et al. Process evaluation of complex interventions: Medical Research Council guidance. BMJ. 2015;350:h1258. Published 2015 Mar 19. doi:10.1136/bmj.h1258

Pallasmaa N, Ekblad U, Gissler M, Alanen A. 2015. The impact of maternal obesity, age, pre-eclampsia and insulin dependent diabetes on severe maternal morbidity by mode of delivery-a register-based cohort study. Arch Gynecol Obstet. 291(2):311-8. doi: 10.1007/s00404-014-3352-z.

Powell, B.J., Waltz, T.J., Chinman, M.J. et al. A refined compilation of implementation strategies: results from the Expert Recommendations for Implementing Change (ERIC) project. Implementation Sci 10, 21 (2015). <https://doi.org/10.1186/s13012-015-0209-1>

Proctor, E.K., Powell, B.J. & McMillen, J.C. Implementation strategies: recommendations for specifying and reporting. *Implementation Sci* **8,** 139 (2013). <https://doi.org/10.1186/1748-5908-8-139>

Saarikko J, Niela-Vilen H, Ekholm E, Hamari L, Azimi I, Liljeberg P, Rahmani AM, Löyttyniemi E, Axelin A. Continuous 7-Month Internet of Things–Based Monitoring of Health Parameters of Pregnant and Postpartum Women: Prospective Observational Feasibility Study. JMIR Form Res JMIR Publications Inc.; 2020 Jul 24 [cited 2020 Dec 21];4(7):e12417. [doi: 10.2196/12417]

TENK 2012. Hyvä tieteellinen käytäntö ja sen loukkausepäilyjen käsitteleminen Suomessa. Tutkimuseettinen neuvottelukunnan ohje 2012. Saatavilla osoitteessa: <http://www.tenk.fi/sites/tenk.fi/files/HTK_ohje_2012.pdf>

THL (Terveyden ja hyvinvoinnin laitos) 2017. Tilastoraportti 37. Perinataalitilasto –synnyttäjät, synnytykset ja vastasyntyneet 2016. <http://www.julkari.fi/bitstream/handle/10024/135445/Tr_37_17.pdf?sequence=1&isAllowed=y> (20.12. 2018)

Weiner, B. J., Lewis, C. C., Stanick, C., Powell, B. J., Dorsey, C. N., Clary, A. S., Boynton, M. H., & Halko, H. (2017). Psychometric assessment of three newly developed implementation outcome measures. Implementation Science, 12(108), 1-12. doi: 10.1186/s13012-017-0635-3

WHO (World Health Organization). 2018. Overweight and obesity factsheet. 16 February 2018. https://www.who.int/en/news-room/fact-sheets/detail/obesity-and-overweight (5.2.2020).

WHOQOL Group. Development of the World Health Organization WHOQOL-BREF quality of life assessment. Psychol Med May. 1998; 28(3):551–558.
