# Supplementary material for: Supporting lifestyle change in obese pregnant mothers through the wearable internet-of-things (SLIM) -intervention for overweight pregnant women: Study protocol for a quasi-experimental trial
Source: PLoS One. 2023 Jan 19;18(1):e0279696. doi: 10.1371/journal.pone.0279696 (PMC9851496; doi:10.1371/journal.pone.0279696)
Supplement: S2 File — (DOCX) [file pone.0279696.s006.docx]

**Development and implementation of Supporting Lifestyle Change in Obese Pregnant Mothers through Wearable Internet-of-Things (SLIM) -intervention for overweight pregnant women**

*Johanna Saarikko, MHSc, doctoral candidate, Department of Nursing Science, University of Turku*

*Hannakaisa Niela-Vilén, PhD, Department of Nursing Science, University of Turku*

*Emilia Huvinen, MD, Department of Obstetrics and Gynaecology, University of Helsinki and Helsinki University Hospital*

*Eeva Ekholm, MD, Department of Obstetrics and Gynecology, Turku University Hospital and University of Turku*

*Iman Azimi, PhD, Department of Future Technologies, University of Turku*

*Fatemeh Sarhaddi, doctoral candidate, Department of Future Technologies, University of Turku*

*Milad Asgari, doctoral candidate, Computer science, University of California, Irvine, USA*

*Amir Rahmani, MBA, PhD, School of Nursing and Department of Computer Science, University of California, Irvine, USA*

*Pasi Liljeberg, PhD, professor, Department of Future Technologies, University of Turku*

*Anna Axelin, PhD, Assistant professor, Department of Nursing Science, University of Turku*

1. **Background**

Obesity (BMI ≥ 30) is globally increasing problem. In industrialized countries, obesity is one of the most significant causes of death and disease (WHO 2018). Obesity is also increasing among pregnant women and is a significant concern in public health care (Ng et al. 2013). In Finland, 36% of pregnant women are overweight (BMI ≥ 25) and 14% are obese (THL 2017). The overweight before and during pregnancy has long-term effects on the health of the unborn child. Maternal overweight increases the risk of, for example, fetal macrosomia, intrapartum complications and ceasarean sections (European Perinatal Health Report 2015). as well as risks of gestational diabetes and pre-eclampsia (Pallasmaa et al. 2015). In addition, offspring of overweight women are more likely to be overweight or obese (Poston 2012).

Prevention of excessive weight gain during pregnancy is important especially for women who are overweight before pregnancy. Previous weight management interventions for pregnant women have reduced weight gain during pregnancy (i-WIP 2017). Weight management interventions have also reduced postpartum depression and weight retention. Interventions may have significant long-term effects on the health of both, mother and her child (Brown et al. 2017). Overweight mothers should also be encouraged to breastfeed, which has been found to reduce the risk of diabetes and cardiovascular diseases. In addition, breastfeeding women have been found to have a decreased risk of obesity later in life. (Dutton et al. 2018; Bobrow et al. 2013.)

Although previous weight management interventions have succeeded in reducing gestational weight, the implementation of interventions is challenging. In our first sub-study (reporting phase), we described the experiences of health care professionals and overweight pregnant women and overweight women who have recently given birth, on the weight management practices in maternity clinics, using a qualitative research design. Utilizing the Behavior of Change Wheel (BCW), the key intervention functions of the weight management intervention were identified from the data: education, persuasion, enablement, environmental restructuring and training. In accordance with the BCW theory, behavior change techniques (Behaviour change techniques v1) were formed: goal setting (behavior), goal setting (outcomes), review behavioral goals, self-monitoring of behavior, adding objects to the environment, information about health consequences, feedback on behavior and outcomes (Michie et al. 2011) Implementation strategies, were classified as Expert Recommendations for Implementing Change (ERIC) compilation (Powell et al. 2015) and Proctor et al. (2013) recommendations.

Health technology can be utilized in intervention implementation. In our previous usability study, pregnant women wore smart wristband, which measured the activity, sleep and heart during pregnancy. Of the seven-month follow-up period, pregnant women wore the smart wristband approximately for five months (Saarikko et al. 2020; Grym et al. 2019). With a remote monitoring system, it would be possible to continuously monitor the physiological parameters, activity and nutrition of the pregnant women as part of an innovative weight management intervention. Further, remote monitoring can probably support the implementation of the intervention. The focus of SLIM intervention is in supporting and refining existing antenatal services.

1. **Objectives**

The purpose of this study is to assess the effectiveness of the SLIM intervention (primary outcome: self-efficacy in eating and physical activity; secondary outcome: weight gain; possibly further modelling of the data) and evaluate the implementation of intervention regarding perceived fidelity, acceptability, appropriateness and feasibility among health professionals in maternity clinics and women with overweight or obesity.

Research questions:

1. What is the effectiveness of the weight management intervention in terms of improving self-efficacy in eating and physical activity during pregnancy and after delivery?

2. What is the effectiveness of the weight management intervention in terms of preventing excessive gestational weight gain, reducing depressive symptoms and increasing quality of life and sense of coherence during pregnancy and after delivery?

3. What is the implementation of intervention regarding perceived fidelity, acceptability, appropriateness?

4. What is the feasibility of the intervention to be used in maternity clinics?

***Hypothesis:*** The self-efficacy of overweight pregnant women increases in women participating in the SLIM intervention during pregnancy and after delivery

1. **Methods**

This is a prospective, non-randomized, quasi-experimental (pre-post) intervention trial including an embedded mixed-method process evaluation. The study will be conducted in two public maternity clinics located in four municipalities in the Hospital District of Southwest Finland between April 2021 and May 2023. The power analysis was calculated based on two scales, self-efficacy in eating (WEL) and physical activity (PASE). Calculations were done with one sided t-tests with effect size 0.50, power level of 0.80 and significance level of 0.05. Loss of 20% was included in the calculation. Based on both scales, a sample of 54 overweight pregnant women was needed to detect the difference in the change in eating self-efficacy or self-efficacy for physical activity. Overweight women (n = 54) will be recruited in their first antenatal visit using convenience sampling.

Women are eligible for inclusion if they are 1) pregnant ≤15 weeks of gestation, 2) ≥18 years of age and 3) overweight or obese (BMI ≥ 25kg/m2) and 4) have adequate language skills in Finnish. Women are excluded if they 1) don’t have a mobile device to download applications and synchronize data, 2) have a diagnosis of severe mental illness, 3) are diagnosed diabetes type I before pregnancy and/or 4) have mobility limitations. All public health nurses working in selected maternity clinics were eligible for the study.

The public health nurses of maternity clinics (n = 9) have already been recruited in the first phase of the study. They are in key position to recruit pregnant women. The public health nurse will provide a letter of information to overweight pregnant women and ask permission for the researchers to be in contact with potential participants. The researchers explain the study purpose and procedures to the women in a scheduled appointment. In addition, an Oura ring, and instructions on how to use it and other applications will be given to each participant by researchers after informed consent.

1. **SLIM Intervention**

The SLIM intervention targets overweight pregnant women with a goal to improve their self-efficacy in weight management. Intervention will be delivered by public health nurses working in maternity clinics starting with the first antenatal appointment (from gestational week 15 or less) continuing with each appointment in maternity clinics until the postnatal appointment 12 weeks after delivery. The core components of the SLIM intervention are health technology, motivational interviewing, feedback and goal setting. Health technology includes Oura ring, electronic food diary and SLIM application. Oura is a light, waterproof smart ring that can be used to measure, for example, heart rate and heart rate variability, steps, activity levels, sleep and body temperature. Oura can be used in any finger in either hand. The electronic food diary, FatSecret is a free of charge online application service that allows users to set up unique user profiles, track their activity, nutrition and weight. The service offered by FatSecret include the FatSecret websites and mobile applications. (Fatsecret 2022.) Participants will fill in a food diary for one week at three time points: gestational week 15 or less, gestational week 34 and eight weeks after the delivery. The SLIM application is a platform for sending the questionnaires and surveys to participants. Public health nurses can utilize the data monitored and collected with Oura smartring and food diary during routine maternity care visits to evaluate physical activity, sleep and nutrition. Public health nurses will use motivational interviewing focusing on helping individuals to identify and resolve ambivalence on changing behavior (Elwyn et al 2014). They will give feedback to women based on data and create goals in collaboration with women. Goals are documented in patient records.

**Data collection**

***Outcome measures***

The primary outcomes for the effectiveness of the intervention are self-efficacy in eating and physical activity and weight gain during pregnancy (secondary outcome). To track variations and trends, health parameters like weight retention postpartum, depressive symptoms, quality of life, pregnancy specific anxiety, perceived stress, sense of coherence, eating behavior and lifestyle patterns, physical activity levels, sleep quality, stress levels and dietary intake are collected.

***Process evaluation of the implementation***

A process evaluation will be conducted using a mixed-method approach to examine the feasibility and acceptability of the intervention, the implementation strategy and study processes including data collection (Craig et al 2013). To enable this evaluation, the primary intervention’s components (i.e., its intervention and delivery strategies), and implementation outcomes (in terms of acceptability, fidelity, mechanism of impact and contextual influences) will be defined with the help of a logic model (Kellogg 2004; Moore et al 2014). To evaluate the coverage of the implementation, the logbook of participant recruitment will be documented. In addition, the level of missing data in returned questionnaires will be assessed. Public health nurses’ perception of appropriateness, acceptability and feasibility of the intervention is evaluated with validated AIM, IAM & FIM questionnaires (Weiner et al 2017) in every six months. In addition, all public health nurses will be invited to take part in focus group exit interviews to assess the fidelity and adoption of the intervention at the end of the study. In addition, to assess fidelity of the intervention, public health nurses will fill a logbook about the content of maternity care appointments related to the SLIM intervention.

***Data analysis***

The data will be analysed statistically. Appropriate descriptive statistics will be calculated for the variables. Mixed effect models will be used to evaluate change over time in self-efficacy in weight management and weight change. Simple mediation models will be used to assess calories consumed and MVPA as mediators between self-efficacy and weight change. Qualitative data, recorded interviews, will be transcribed verbatim and transferred to NVivo. Data will be analysed using thematic analysis (Braun & Clarke 2006). Qualitative and quantitative data will be integrated as a part of process evaluation. The change in AIM, IAM and FIM will be analysed between pregnancy week 34 and 12 weeks after birth, using paired t-tests or Wilcoxon signed rank tests.

1. **Ethical considerations**

The study will be conducted in accordance with the Helsinki Declaration. The statement will be applied by the Joint Ethics Committee of the Hospital District of Southwest Finland and permission by the maternity clinics. The study will comply with the act (488/99) and the degree (986/99) on medical research and the act on the status and rights of patients (785/92). The transfer of personal data outside the EU and EEA is based on standard contractual clauses (SCC) approved by the Commission between the controller (University of Turku) and the personal data processor (UCI). Throughout the research, good scientific practice is followed according to the recommendations of the Research Ethics Advisory Board (TENK 2012). As an additional protection measure for the processing of personal data, an impact assessment has been carried out. The participants are given information orally and written, and they are reminded that participation is voluntary. Pregnant women are particularly vulnerable group of participants, but similar results cannot be obtained with other participants. The research does not cause additional visits in maternity care or incur costs. Participants will be asked for written informed consent to participate in the study. Those recruited into the study are also given time to consider their consent if necessary.

The collected data will be managed and stored securely and will not be available to anyone outside the research group. In addition, the data and the computers are stored in appropriately locked rooms. All data is processed in pseudonymized form. The University of Turku is responsible for the legality of personal data processing. The data will be destroyed five years after the publication of the results. With the collected data, it is possible to get information about the factors influencing the implementation of the weight management intervention. The information will be used in future in the wider implementation of the intervention. The benefits obtained from the research clearly outweigh the harms arising from it.

**References**

Antonovsky, A. 1987. Unraveling the mystery of health. How people manage stress and stay well. San Francisco: Jossey-Bass Publishers

Asetus lääketieteellisestä tutkimuksesta 986/1999. <https://www.finlex.fi/fi/laki/alkup/1999/19990986>

Brown J., Alwan N.A., West J., Brown S., McKinlay C.J.D., Farrar D., Crowther C.A. 2017. Lifestyle interventions for the treatment of women with gestational diabetes (Review). Cochrane Database Syst Rev. 4(5). CD011970. doi: 10.1002/14651858.CD011970.pub2.

Bobrow KL, Quigley MA, Green J, Reeves GK, Beral V for the Million Women Study Collaborators. 2013. Persistent effects of women’s parity and breastfeeding patterns on their body mass index: results from the Million Women Study. Int J Obes (Lond). 37(5):712-7. doi: 10.1038/ijo.2012.76.

Clark, M. M., Abrams, D. B., Niaura, R. S., Eaton, C. A., & Rossi, J. S. (1991). Self-efficacy in weight management. Journal of Consulting and Clinical Psychology, 59(5), 739–744. <https://doi.org/10.1037/0022-006X.59.5.739>

Clark, M.M., Cargill, B.R., Medeiros, M.L., Pera, V. 1996. Changes in Self-Efficacy Following Obesity Treatment.Obesity research 1996 ; 2(4) : 179-181.

Cohen S, Kamarck T, Mermelstein R. .1983 A global measure of perceived stress. Journal of Health and Social Behavior Vol. 24, No. 4, pp. 385-396

Cox, J.L., Holden, J.M., and Sagovsky, R. 1987. Detection of postnatal depression: Development of the 10-item Edinburgh Postnatal Depression Scale. British Journal of Psychiatry 150:782–6.

Dutton H, Borengasser SJ, Gaudet LM, Barbour LA, Keely EJ. 2018. Obesity in Pregnancy: Optimizing Outcomes for Mom and Baby. Med Clin North Am. 102(1), 87-106.

European commission 5.2.2010. Standard Contractual Clauses (SCC) Standard contractual clauses for data transfers between EU and non-EUcountries.

<https://ec.europa.eu/info/law/law-topic/data-protection/international-dimension-data-protection/standard-contractual-clauses-scc_en>

European Perinatal Health Report 2015. Core indicators of the health and care of pregnant women and babies in Europe in 2015. November 2018. <https://www.europeristat.com/images/EPHR2015_web_hyperlinked_Euro-Peristat.pdf> (21.12.2020).

Grol R., Wensing M., Eccles M., Davis D. (toim.) 2013. Improving Patient Care. The implementation of change in health care. Second edition. UK: Wiley Blackwell.

Grym K, Niela-Vilén H, Ekholm E, Hamari L, Azimi I, Rahmani A, et al. Feasibility of smart wristbands for continuous monitoring during pregnancy and one month after birth. BMC Pregnancy Childbirth 2019 Jan 17;19(1):34 [FREE Full text] [doi: 10.1186/s12884-019-2187-9] [Medline: 30654747]

Huizink AC, Delforterie MJ, Scheinin NM, Tolvanen M, Karlsson L, Karlsson H. Adaption of pregnancy anxiety questionnaire-revised for all pregnant women regardless of parity: PRAQ-R2. Arch Womens Ment Health. 2016;19(1):125-132. doi:10.1007/s00737-015-0531-2

i-WIP (The International Weight Management in Pregnancy) Collaborative Group. 2017. Effect of diet and physical activity based interventions in pregnancy on gestational weight gain and pregnancy outcomes: meta-analysis of individual participant data from randomised trials. BMJ 19(358) 3119. doi: 10.1136/bmj.j3119

Johansson S, Villamor E, Altman M, Bonamy AK, Granath F, Cnattingius S, 2014. Maternal overweight and obesity in early pregnancy and risk of infant mortality: a population based cohort study in Sweden. BMJ 2; 349: g6572. doi: 10.1136/bmj.g6572.

Karlsson J, Persson L-O, Sjöström L, Sullivan M. Psychometric properties and factor structure of the Three-Factor Eating Questionnaire (TFEQ) in obese men and women. Results from the Swedish Obese Subjects (SOS) study. Int J Obes 2000;12:1715-1725.

Kushner RF, Choi SW, Burns JL. Development of a six-factor questionnaire for use in weight management counseling. Patient Educ Couns 2016;99:2018-2025

Laki lääketieteellisestä tutkimuksesta 9.4.1999/488. <https://www.finlex.fi/fi/laki/ajantasa/1999/19990488>

Laki potilaan asemasta ja oikeuksista 17.8.1992/785. <https://www.finlex.fi/fi/laki/ajantasa/1992/19920785>

Marcus BH, Selby VC, Niaura RS, Rossi JS. Self-efficacy and the stages of exercise behavior-change. Res Q Exerc Sport. 1992;63:60–6.

Michie S, van Stralen MM, West R. 2011. The behaviour change wheel: A new method for characterising and designing behaviour change interventions. Implement Sci. 23; 6:42. doi: 10.1186/1748-5908-6-42.

Moore GF, Audrey S, Barker M, et al. Process evaluation of complex interventions: Medical Research Council guidance. BMJ. 2015;350:h1258. Published 2015 Mar 19. doi:10.1136/bmj.h1258

Pallasmaa N, Ekblad U, Gissler M, Alanen A. 2015. The impact of maternal obesity, age, pre-eclampsia and insulin dependent diabetes on severe maternal morbidity by mode of delivery-a register-based cohort study. Arch Gynecol Obstet. 291(2):311-8. doi: 10.1007/s00404-014-3352-z.

Powell, B.J., Waltz, T.J., Chinman, M.J. et al. A refined compilation of implementation strategies: results from the Expert Recommendations for Implementing Change (ERIC) project. Implementation Sci 10, 21 (2015). <https://doi.org/10.1186/s13012-015-0209-1>

Proctor, E.K., Powell, B.J. & McMillen, J.C. Implementation strategies: recommendations for specifying and reporting. *Implementation Sci* **8,** 139 (2013). <https://doi.org/10.1186/1748-5908-8-139>

Saarikko J, Niela-Vilen H, Ekholm E, Hamari L, Azimi I, Liljeberg P, Rahmani AM, Löyttyniemi E, Axelin A. Continuous 7-Month Internet of Things–Based Monitoring of Health Parameters of Pregnant and Postpartum Women: Prospective Observational Feasibility Study. JMIR Form Res JMIR Publications Inc.; 2020 Jul 24 [cited 2020 Dec 21];4(7):e12417. [doi: 10.2196/12417]

TENK 2012. Hyvä tieteellinen käytäntö ja sen loukkausepäilyjen käsitteleminen Suomessa. Tutkimuseettinen neuvottelukunnan ohje 2012. Saatavilla osoitteessa: <http://www.tenk.fi/sites/tenk.fi/files/HTK_ohje_2012.pdf>

THL (Terveyden ja hyvinvoinnin laitos) 2017. Tilastoraportti 37. Perinataalitilasto –synnyttäjät, synnytykset ja vastasyntyneet 2016. <http://www.julkari.fi/bitstream/handle/10024/135445/Tr_37_17.pdf?sequence=1&isAllowed=y> (20.12. 2018)

Weiner, B. J., Lewis, C. C., Stanick, C., Powell, B. J., Dorsey, C. N., Clary, A. S., Boynton, M. H., & Halko, H. (2017). Psychometric assessment of three newly developed implementation outcome measures. Implementation Science, 12(108), 1-12. doi: 10.1186/s13012-017-0635-3

WHO (World Health Organization). 2018. Overweight and obesity factsheet. 16 February 2018. https://www.who.int/en/news-room/fact-sheets/detail/obesity-and-overweight (5.2.2020).

WHOQOL Group. Development of the World Health Organization WHOQOL-BREF quality of life assessment. Psychol Med May. 1998; 28(3):551–558.
